# Transcriptomic Analysis of Leaf in Tree Peony Reveals Differentially Expressed Pigments Genes

**DOI:** 10.3390/molecules22020324

**Published:** 2017-02-20

**Authors:** Jianrang Luo, Qianqian Shi, Lixin Niu, Yanlong Zhang

**Affiliations:** College of Landscape Architecture and Art, Northwest A & F University, Yangling 712100, Shaanxi, China; luojianrang@163.com (J.L.); shiqianqian2005@163.com (Q.S.)

**Keywords:** tree peony, transcriptome, flavonoids, chlorophyll, carotenoid, differentially expressed genes (DEGs)

## Abstract

Tree peony (*Paeonia suffruticosa* Andrews) is an important traditional flower in China. Besides its beautiful flower, the leaf of tree peony has also good ornamental value owing to its leaf color change in spring. So far, the molecular mechanism of leaf color change in tree peony is unclear. In this study, the pigment level and transcriptome of three different color stages of tree peony leaf were analyzed. The purplish red leaf was rich in anthocyanin, while yellowish green leaf was rich in chlorophyll and carotenoid. Transcriptome analysis revealed that 4302 differentially expressed genes (DEGs) were upregulated, and 4225 were downregulated in the purplish red leaf vs. yellowish green leaf. Among these DEGs, eight genes were predicted to participate in anthocyanin biosynthesis, eight genes were predicted involved in porphyrin and chlorophyll metabolism, and 10 genes were predicted to participate in carotenoid metabolism. In addition, 27 MYBs, 20 bHLHs, 36 WD40 genes were also identified from DEGs. Anthocyanidin synthase (*ANS*) is the key gene that controls the anthocyanin level in tree peony leaf. Protochlorophyllide oxido-reductase (*POR*) is the key gene which regulated the chlorophyll content in tree peony leaf.

## 1. Introduction

Tree peony (*Paeonia suffruticosa* Andr.) is a woody shrub of the genus *Paeonia* and family Paeoniaceae, and is a traditional ornamental flower because of its large, showy, and colorful flowers [1]. Besides its beautiful flower, the leaf of tree peony has also good ornamental value owing to its leaf color change in spring. 

Coloration of plant organs such as flowers, fruit and leaves is governed by pigments in plants [2]. There are four main classes of pigments for coloration in plants: flavonoids/anthocyanins, chlorophylls, carotenoids, and betalains [3]. Flavonoids, particularly anthocyanins, acting as major pigments that responsible for the orange to blue colors found in many plant flowers, leaves, fruits, seeds, and other tissues [2,3,4]. Chlorophylls including chlorophyll a and chlorophyll b, the photosynthetic pigments that play essential roles in plant photosynthesis, are main pigments in plant green leaf [5]. Carotenoid belong to the isoprenoid pigments, which are usually responsible for yellow-orange colors [6,7]. Carotenoids are also photosynthetic pigments that usually exist in plant leaf with chlorophylls. Betalains are the yellow and violet pigments belong to the order Caryophyllales, and they only exist in very few plants such as *Beta vulgaris* and *Suaeda salsa* [8,9]. In plants, anthocyanins and betalains are usually mutual exclusive, which are never found together in the same plant [10].

Prior to advancements in this field, researchers mainly focused on floral flavonoid/anthocyanin pigment components detection [11,12,13], or flavonoid/anthocyanin genes of petal analysis in tree peony [14,15,16,17]. As far as we know, there has been no report on leaf pigments components detection and related gene analysis in tree peony. In the absence of a complete genome sequence in tree peony, transcriptomic analysis is an effective method for gaining insights into differentially expressed genes (DEGs) between different color leaves. Transcriptome sequencing of the different leaf color stages of tree peony will provide useful insights into the molecular mechanisms of tree peony leaf color changes in spring. Therefore, the leaf pigment levels of different stages in tree peony was analyzed. The different leaf color stage transcriptomes were compared, and the DEGs were filtered. Our main objective was to annotate and analyze the DEGs involved in flavonoid/anthocyanin biosynthesis, chlorophyll and carotenoid biosynthesis to identify the candidate genes responsible for tree peony purplish red leaf in spring.

## 2. Results

### 2.1. Pigments Level in the Leaf of Three Developmental Stages in Tree Peony

**Figure 1:**
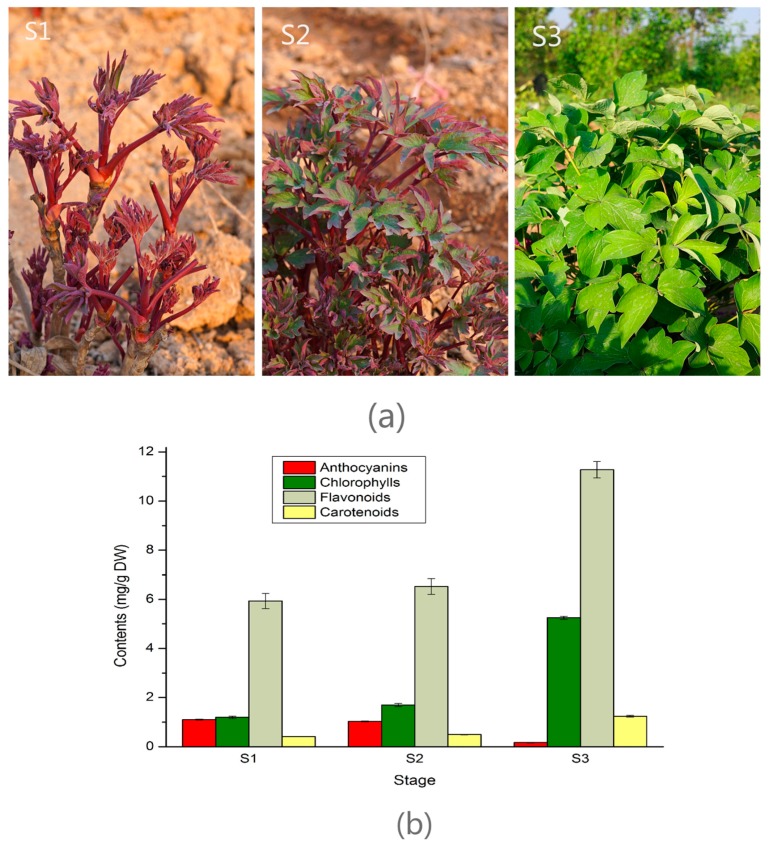
Images of tree peony leaf and pigments contents at three stages. (**a**) Images of tree peony leaf at three different stages; (**b**) contents of anthocyanin, chlorophyll, total flavonoid, and carotenoid in the leaf of three different stages.

In spring, as the tree peony leaf developed two major physiological changes, color fading and yellowish greening were visually apparent (Figure 1a). The tree peony leaf color was purplish red at S1, then changed to half purplish red and half yellowish green at S2, finally yellowish green at S3. As shown in Figure 1b, the anthocyanin contents were decreased from S1 to S3. While the total flavonoid, carotenoid, and chlorophyll contents were increased from S1 to S3. These indicated that the change in leaf color phenotypes of tree peony ‘Man Yuan Chun Guang’ was mainly caused by the decrease of anthocyanin content and increase of chlorophyll and carotenoid.

### 2.2. Library Construction and Transcriptome Sequencing

To understand the molecular basis of leaf color change in tree peony, the leaves of S1, S2, and S3 were used to build three libraries for high-throughput sequencing. 64,530,684; 67,008,409; and 69,283,538 raw reads were obtained from S1, S2, and S3 sequencing libraries, respectively. After removal of adaptor sequences, ambiguous reads, and low-quality reads, 62,835,627; 65,363,790; and 67,681,919 high quality clean reads comprising 9.43, 9.80,10.15 Gb nucleotides with Q20 > 98% were generated from S1, S2, and S3 transcriptome sequencing. In total, 53,323 unigenes with an N50 of 1215 were generated byTrinity 2.1.1 (Broad Institute, Cambridge, MA, USA). The min length, max length and average length of these unigenes were 201, 13121, and 801 nt, respectively. The size distributions of these unigenes are shown in Supplementary Materials Figure S1.

### 2.3. De Novo Assembly and Gene Annotation

**Figure 2:**
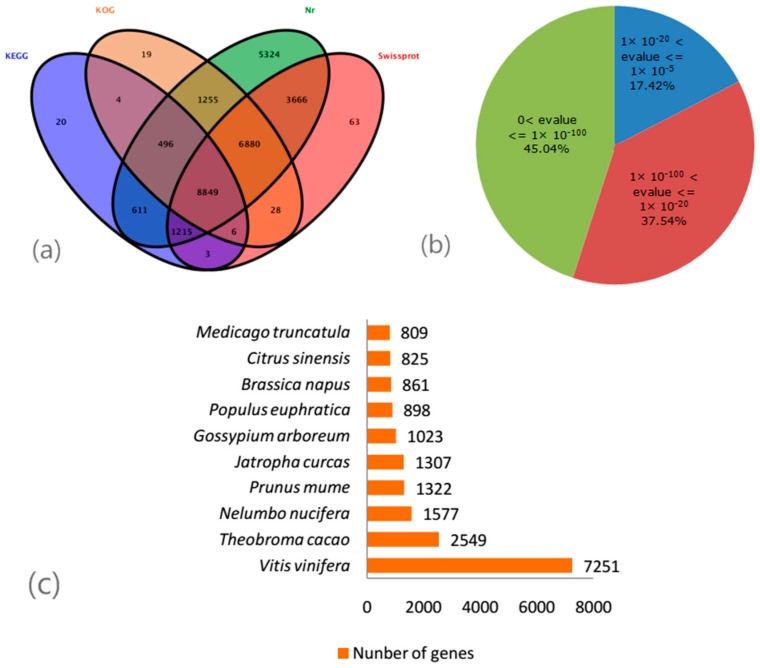
Outcome of homology search of tree peony unigenes against the Nr database. (**a**): Venn diagram of unigene numbers annotated by BLASTx with an E-value threshold of 1 × 10^−5^ against protein databases. The numbers in the circles indicate unigenes numbers annotated by single or multiple databases; (**b**): E-value distribution of the top BLAST hits for each unigene sequence; (**c**): Species distribution of the top BLAST hits for all homologous sequences.

A total of 28,439 unigenes (53.33% of all 53,323 cleaned unigenes) were annotated based on BLAST+ 2.4.0 (http://www.ncbi.nlm.nih.gov/BLAST/) (E-value < 1 × 10^−5^) searches of four public databases. Among them, 28,296 unigenes (53.06%); 20,710 (38.84%); 17,537 (32.89%); 11,204 (21.01%) could be annotated to the NCBI nr database released 31 August 2016 (http://www.ncbi.nlm.nih.gov), the Swiss-Prot protein database (http://www.expasy.ch/sprot), the COG/KOG databases (http://www.ncbi.nlm.nih.gov/COG), and the KEGG database (http://www.genome.jp/kegg), respectively (Figure 2a). Based on the NCBI nr annotations, 45.04% of the annotated sequences had very strong homology (E-value ≤ 1 × 10^−100^), 37.54% showed strong homology (1 × 10^−100^ < E-value ≤ 1 × 10^−20^), an additional 17.42% showed homology (1 × 10^−20^ < E-value ≤ 1 × 10^−5^) to available plant sequences (Figure 2b). 7251 unique sequences had top matches to sequences from *Vitis vinifera*, and followed by *Theobroma cacao* (2549), *Nelumbo nucifera* (1577), *Prunus mume* (1322), *Jatropha curcas* (1307), *Gossypium arboreum* (1023), *Populus euphratica* (898), *Brassica napus* (861), *Citrus sinensis* (825), and *Medicago truncatula* (809) (Figure 2c).

### 2.4. Identification and Analysis of DEGs between Three Leaf Color Stages

**Figure 3:**
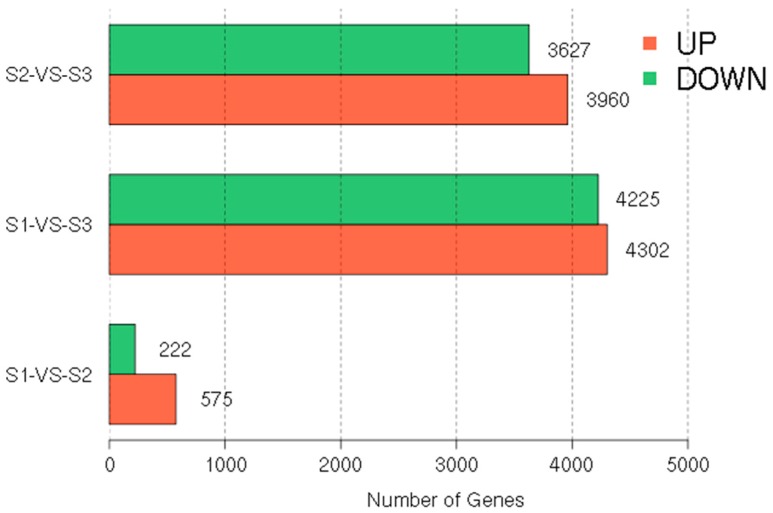
Differential gene expression profiles based on the library of the three leaf stages.

The thresholds false discovery rate (FDR) < 0.05 and absolute value of log2 ratio > 1 were used to judge the significance of the DEGs at three leaf color stages (S1 vs. S2, S2 vs. S3, S1 vs. S3). There were 797 DEGs between S1 and S2 libraries, in which 575 unigenes were significantly upregulated and 222 unigenes were downregulated. A total of 7587 DEGs were found between S2 and S3 libraries, with 3627 downregulated and 3960 upregulated. Finally, the greatest number of differentially expressed genes (8527 DEGs) occurred between S1 and S3 libraries, with 4225 downregulated and 4302 upregulated (Figure 3). Therefore, DEGs between S1 and S3 libraries were used for further analysis.

Among those differentially expressed genes with a KEGG pathway annotation released 1 July 2016 (http://www.genome.jp/kegg), 1436 DEGs in S1 vs. S3 library were mapped to 121 pathways (Table S1). Since plant leaf pigment was mainly correlated with the accumulation of anthocyanin, chlorophyll, and carotenoid, we were interested in these pigments biosynthesis pathway. Seven pathways including ‘phenylpropanoid biosynthesis’ (44 DEGs, ko00940), ‘flavonoid biosynthesis’ (8 DEGs, ko00941), ‘flavone and flavonol biosynthesis’ (1 DEGs, ko00944), ‘anthocyanin biosynthesis’ (2 DEGs, ko00942), ‘carotenoid biosynthesis’ (13 DEGs, ko00906), ‘isoflavonoid biosynthesis’ (3 DEGs, ko00943), and ‘porphyrin and chlorophyll metabolism’ (4 DEGs, ko00860) were identified which were related to plant organ color development. In addition, there were six glucide metabolic pathways, including ‘starch and sucrose metabolism’ (63 DEGs, ko00500), ‘glycosaminoglycan degradation’ (1 DEG, ko00531), ‘amino sugar and nucleotide sugar metabolism’ (28 DEGs, ko00520), ‘pentose and glucuronate interconversions’ (21 DEGs, ko00040), ‘fructose and mannose metabolism’ (23 DEGs, ko00051), and ‘glycolysis/gluconeogenesis’ (42 DEGs, ko00010). All of these metabolic pathways may relate to the synthesis of substrates for anthocyanin biosynthesis.

### 2.5. Annotation of DEGs Probably Involved in Flavonoid/Anthocyanin Biosynthesis

By annotation in public database, eight DEGs were predicted to participate in flavonoid/anthocyanin biosynthesis pathway (Table 1). Among them, Unigene0034354, Unigene0040930, Unigene0046696, and Unigene0028008 were annotated as flavanone 3-hydroxylase (*F3H*), flavonoid 3′-hydroxylase (*F3′H*), dihydroflavonol 4-reductase (*DFR*), and anthocyanidin synthase (*ANS*), respectively. They were the same genes with that reported previously in tree peony [14]. Unigene0004270 has 59% amino acid similarity with chalcone isomerase (*CHI*) (JN119872) in herbaceous peony, Unigene0018412 was annotated as hydroxycinnamoyl transferase (*HCT*), encoding HCT which catalyze p-coumaroyl-CoA into caffeoyl-CoA that is continually converted to eriodictyol (a flavanone). Unigene0036137 and Unigene0052871 were annotated as UDP-glycosyltransferase and glucoside glucosyltransferase, these two genes were related with anthocyanidin glycosylation.

In anthocyanin biosynthesis pathway, the spatial and temporal expression of structural genes such as *F3H*, *DFR*, and *ANS* is usually controlled by transcription factors from MYB, bHLH, and WD40 families [18,19,20,21]. In our study 27 MYBs, 20 bHLHs, and 36 WD40 were identified from DEGs (Table 2). Among these transcription factors, 15 MYBs, 15 bHLHs, and 29 WD40 were upregulated in purplish red leaf. It indicated that these upregulation transcription factors were positively related with anthocyanin level in tree peony leaf.

### 2.6. Annotation of DEGs Probably Involved in Chlorophyll Biosynthesis

Four DEGs involved in porphyrin and chlorophyll metabolism between S1 and S3 were identified, while in S2 vs. S3, eight DEGs involved in porphyrin and chlorophyll metabolism were found. Therefore, these eight DEGs involved in porphyrin and chlorophyll metabolism in S2 vs. S3 were used for further analysis (Table 3). As shown in Table 3, Unigene0031269, Unigene0022913, Unigene0032398, Unigene0036679, Unigene0026448, and Unigene0047139 showed higher transcript abundance in S3 compared with S2, while the expression level of Unigene0045551 and Unigene0039869 were lower in S3 than in S2. It implied that the expression of Unigene0031269, Unigene0022913, Unigene0032398, Unigene0036679, Unigene0026448, and Unigene0047139 have a positive role in chlorophyll accumulation. According to the public database, Unigene0031269, Unigene0022913, Unigene0032398, Unigene0036679, Unigene0026448, and Unigene0047139 were annotated as Mg-protoporphyrinogen IX monomethylester cyclase gene (*CRD1*), divinyl reductase gene (*DVR*), protochlorophyllide oxidoreductase gene (*POR*) [22], chlorophyllide a oxygenase gene (*CAO*), protoheme ferro-lyase gene (*HEMH*), and pheophorbide a oxygenase gene (*PAO*).

### 2.7. Annotation of DEGs Probably Involved in Carotenoid Biosynthesis

**Figure 4:**
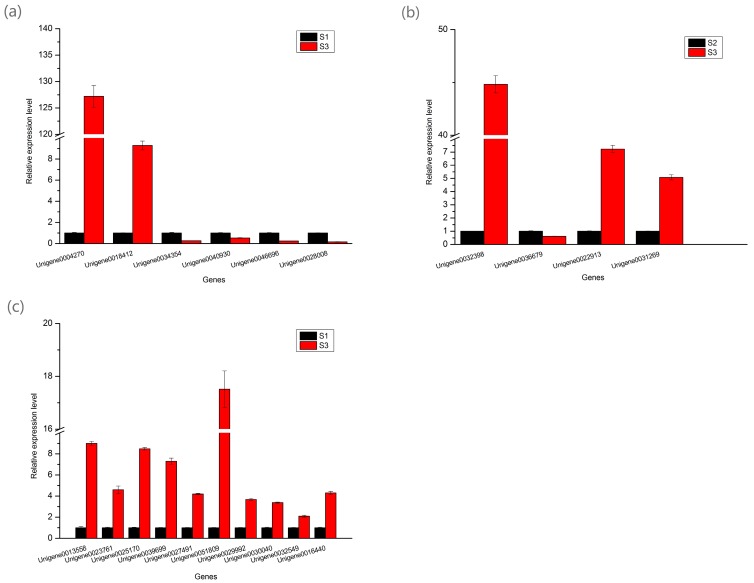
The expression patterns of DEGs involved in anthocyanin (**a**), chlorophyll (**b**), and carotenoid (**c**) biosynthesis revealed by real-time quantitative PCR.

In addition, 10 DEGs were predicted to participate in carotenoid biosynthesis metabolism by annotation in public database (Table 4). Unigene0029992, Unigene0030040, Unigene0039699, Unigene0025170, Unigene0013558, Unigene0023761, Unigene0016440, Unigene0032549, Unigene0027491, and Unigene0051809 were annotated as phytoene synthase (*PSY*), phytoene desaturase (*PDS*), ζ-carotene desaturase (*ZDS*), β-carotene isomerase (*B-ISO*), violaxanthin de-epoxidase (*VDE*), lycopene β cyclase (*LCYB*), lycopene epsilon cyclase (*LCYE*), β-carotene hydroxylase (*CHYB*), zeaxanthin epoxidase (*ZEP*), and carotenoid cleavage dioxygenase (*NECD*) [7,23,24]. All of these genes were upregulated in S3 stage than in S1 according to RNA-Seq data. In real-time quantitative PCR, these 10 Unigenes were also expressed higher in S3 than in S1 (Figure 4c).

### 2.8. Real-Time Quantitative PCR Analysis of the DEGs Involved in Pigment Biosynthesis

To test the reliability of the RNA-Seq data, 20 DEGs participated in tree peony leaf pigments biosynthesis including 6 DEGs involved in anthocyanin biosynthesis, 4 DEGs involved in chlorophyll biosynthesis, and 10 DEGs involved in carotenoid biosynthesis were selected for real-time quantitative PCR (q-PCR) analysis. Unigene0020903 (ubiquitin) was used as internal control. The expression patterns revealed by q-PCR analysis were similar to those revealed by RNA-Seq for the same genes (Figure 4).

## 3. Discussion

Non-green leaves occur in many plants which are very popular in modern landscape garden with its unique ornamental values. Prior to the previous reports, there are three main pigments responsible for non-green leaves. Anthocyanidin accumulation was a major reason for the formation of red color leaves [25,26,27]. Carotenoid was usually responsible for the formation of yellow color [6,28]. Chlorophyll is mainly responsible for the formation of green color [28,29]. High concentrations of chlorophylls can mask the red or yellow color that is provided by anthocyanin or carotenoid [29,30]. In the present study, leaf color altered from purplish red to yellowish green complied with leaf development of tree peony. The anthocyanidins level of the purplish red leaf was significantly higher than that of yellowish green leaf, while chlorophylls and carotenoid level of purplish red leaf was significantly lower than that of the yellowish green leaf. These results indicated that purplish red leaf in tree peony could be attributed to anthocyanidin accumulation. The decrease of anthocyanidin, the increase of chlorophyll and carotenoid were mainly responsible for the alteration from purplish red to yellowish green of tree peony leaf in spring.

According to RNA-Seq data, four anthocyanin structural genes including *F3H, F3’H, DFR* and *ANS* were upregulated in the purplish red leaf and downregulated in the yellowish green leaf. While another two anthocyanin structural genes (*CHI* and *HCT*) were downregulated in the purplish red leaf and upregulated in the yellowish green leaf. Which were similar to those revealed by q-PCR analysis for the same genes. CHI is the enzyme that catalyze 2′,4′,4′,6′-tetrahydroxy chalcone form to naringenin (a flavanone). HCT can catalyze p-Coumaroyl-CoA produce Caffeoyl-CoA, then synthesize eriodictyol (a flavanone). F3H belongs to the OGD family, which catalyzes naringenin yield dihydrokaempferol. Dihydrokaempferol is catalyzed by F3’H to produce dihydroquercetin [3]. Dihydrokaempferol and dihydroquercetin are catalyzed by DFR to form leucoanthocyanidins, and then generate colored anthocyanidins catalyzed by *ANS* [3]. High expression of *CHI* and *HCT* can produced more flavones, while anthocyanin production can be restrained by low expression of *F3H*, *F3′H*, *DFR*, and *ANS*. Owing to higher expression of *CHI*, *HCT*, and lower expression of *F3H*, *F3′H*, *DFR*, *ANS* in yellowish green leaf caused the higher accumulation of non-anthocyanin flavonoids (flavones or flavonols), which was verified by leaf pigment level detection (Figure 2). The pigment level analysis showed that anthocyanin content in leaf was consistent with the trend of leaf color variance. Therefore, co-expression of *F3H*, *F3′H*, *DFR*, and *ANS* was responsible for red pigmentation in the leaf of *Paeonia suffruticosa* ‘Man Yuan Chun Guang’. 

*CHI* is an upstream gene in anthocyanin biosynthesis that controls conversion of chalcones to (2*S*)-naringenin [3]. In this study, we found that *CHI* showed very higher expression level in yellowish green leaf stage compared with purplish red leaf stage. In *P. suffruticosa* ‘Luoyang Hong’, the expression level of *F3H*, *F3’H*, *DFR*, and *ANS* was higher in red petal, while the expression level of *CHI* in red petal was the lowest [14]. The similar expression pattern of *CHI* was also found in *P. lactiflora* [31]. While in the leaf of *Ginkgo biloba*, an organ lacking anthocyanin, the highly expression level of *CHI* was detected [32]. Li et al. (2006) reported that transgenic tobacco plants over-expressing sense Asteraceae *CHI* appeared normal in flower color along with an elevated accumulation of flavonoids, but not anthocyanin [33]. In addition, over-expression of *CHI* from tree peony can increased the level of total flavones and flavonols but reduced the intensity of flower pigmentation in transgenic tobacco [16]. The expression of *CHI* not only involved in anthocyanin biosynthesis, but also other secondary metabolites biosynthesis, such as flavones and flavonols [3]. All of these indicted that *CHI* is the key gene affecting the accumulation of flavonoids in plant, but not anthocyanin.

*ANS* is a downstream gene in anthocyanin biosynthesis, which encoded anthocyanidin synthase that catalyzes the oxidation of the colorless leucoanthocyanidin to the corresponding colored anthocyanidin [3]. In the present study, the expression level of *ANS* was 6.43-fold higher in purplish red leaf vs. yellowish green leaf, which was significantly higher than other three candidate structural genes of anthocyanin biosynthesis (Figure 4a), suggesting that *ANS* could be the key gene of anthocyanin accumulation in tree peony leaf. In herbaceous peony, *ANS* had the highest levels in red flower cultivar and the lowest levels in white flower cultivar, which resulted in the shift from white to pink and red in flowers [4]. In five cultivars of sweet potato, *ANS* expression was strongly associated with anthocyanin accumulation [34]. The similar result was also found in *Chrysanthemum grandiflorum* [35] and *Malus* sp. [36]. In addition, the silencing of *ANS* in red colored apple cultivar caused the shift of red leaf to green leaf, suggesting that anthocyanin biosynthesis was almost completely blocked [37]. All these implied that *ANS* was the key gene affecting the accumulation of anthocyanin in plant.

In our present study, eight DEGs (Unigene0045551, Unigene0031269, Unigene0022913, Unigene0032398, Unigene0036679, Unigene0039869, Unigene0026448, and Unigene0047139) potentially involved in porphyrin and chlorophyll biosynthesis were found and analyzed (Table 3). Among these genes, Unigene0031269, Unigene0022913, Unigene0032398, Unigene0036679, Unigene0026448, and Unigene0047139 showed higher transcript abundance in green leaf which has higher chlorophyll level compared with purplish red leaf (Table 3). It seemed that these six genes play positive role in chlorophyll accumulation in tree peony leaf. However, Unigene0026448 was annotated as *HEMH*, which encoded protoheme ferro-lyase that catalyzed protoporphyrin IX into protoheme, which will cause the decrease of protoporphyrin IX, an important intermediate product of chlorophyll biosynthesis [38]. Unigene0047139 was annotated as *PAO*, which will cause the opening of the chlorine macrocycle present in chlorophyll, and finally chlorophyll is broken down to colorless linear tetrapyrroles [29,39]. Therefore, Unigene0026448 and Unigene0047139 were not likely the key genes that affect chlorophyll level in tree peony leaf. Unigene0031269 (*CRD1*) encoded Mg-protoporphyrinogen IX monomethylester cyclase that catalyze Mg-protoporphyrin IX monomethylester formed into divinyl protochlorophyllide. Unigene0022913 (*DVR*) encoded divinyl reductase which catalyze divinyl protochlorophyllide produced protochlorophyllide. Unigene0032398 encoded protochlorophyllide oxidoreductase which can catalyze protochlorophyllide into chlorophyllide a or catalyze divinyl protochlorophyllide into divinyl chlorophyllide a depending on the availability of substrates [38]. Unigene0036679 (*CAO*) encoded chlorophyllide a oxygenase which catalyze chlorophyllide a formed to chlorophyllide b. q-PCR data shows that Unigene0031269, Unigene0022913, and Unigene0032398 were expressed higher level in the green leaf compared with the purplish red leaf, especially Unigene0032398 (*POR*) was expressed very highly level (44.82-fold higher) in the green leaf (Figure 4b). These indicated that *POR* was the key gene affecting chlorophyll level in tree peony leaf.

In the present study, 10 DEGs were predicted to participate in carotenoid biosynthesis metabolism in the tree peony leaf (Table 4). According to RNA-Seq data (Table 4) and q-PCR (Figure 4c), these 10 DEGs were all expressed higher in S3 stage which has higher carotenoid content than in S1. It seemed that these genes all play positive role in carotenoid accumulation in tree peony leaf. However, Unigene0051809 was annotated as carotenoid cleavage dioxygenase gene that can coded 9-cisepoxycarotenoid dioxygenase (NCED), which catalyze 9-cis-violaxanthin and 9-cis-neoxanthin cleaved to xanthoxin, the precursor of abscisic acid, (ABA) [3], thus caused the decrease of carotenoid accumulation. Therefore, Unigene0051809 (*NCED*) was not likely the key gene that affect carotenoid level in tree peony leaf.

## 4. Materials and Methods 

### 4.1. Plant Material

*Paeonia suffruticosa* Andr. ‘Man Yuan Chun Guang’ plants were grown under field conditions in Northwest A&F University, Yangling Shaanxi, China. Leaf samples were collected at three different leaf color stages (S1, S2, S3) in the morning during the March and April in 2016 (Figure 1a). In order to avoid the effect of other organisms (arthropods, bacteria, fungi, etc.) on gene expression, only healthy, undiseased and non pest injured leaves were collected. All samples were collected from the same plant and washed three times with distilled water, and immediately frozen in liquid nitrogen, then stored at −80 °C for RNA extraction, and flavonoid/anthocyanin, chlorophyll, and carotenoid analysis. 

### 4.2. Measurement of Leaf Pigments

#### 4.2.1. Anthocyanin Level Measurement 

Freeze-dried leaves were finely ground and 0.5 g was extracted with 15 mL acidic methanol (0.1% hydrochloric acid) at 4 °C in darkness for 24 h, then suspended by ultrasound for 1 h. After centrifugation at 5000 rpm for 1 min, the supernatant was filtered using a 0.22 μm membrane filter. The absorbance was read at 530 nm using a UV–Vis spectrophotometer (UV-3802, Unico, Suite E Dayton, OH, USA). A standard calibration curve was plotted using cyanidin-3-*O*-glucoside (Cy3G). The values were normalized to the dry weight of each sample to indicate anthocyanin level. 

#### 4.2.2. Flavonoid Level Measurement 

Freeze-dried leaves were finely ground and 0.5 g was extracted with 15 mL methanol at 4 °C in darkness for 24 h, then suspended by ultrasound for 1 h. After centrifugation at 5000 rpm for 1 min, the supernatant was filtered using a 0.22 µm membrane filter. 2 mL supernatant, 2 mL 1.5% AlCl_3_, 3 mL 1 M Acetic acid sodium acetate (pH 5.0) were mixed and keep the volume to 10 mL. After incubation for 10 min, the absorbance was read at 415 nm using a UV-Vis spectrophotometer. A standard calibration curve was plotted using rutin. The values were normalized to the dry weight of each sample to indicate total flavonoid level.

#### 4.2.3. Carotenoid Level Measurement

Freeze-dried leaves were finely ground and 0.5 g was extracted with 15 mL petroleum ether at 4 °C in darkness for 24 h, then suspended by ultrasound for 1 h. After centrifugation at 5000 rpm for 1 min, the supernatant was filtered using a 0.22 µm membrane filter. The absorbance was read at 440 nm using a UV-Vis spectrophotometer. A standard calibration curve was plotted using β-caroteneas. The values were normalized to the dry weight of each sample to indicate carotenoid level. 

#### 4.2.4. Chlorophyll Level Measurement

Freeze-dried leaves were finely ground and 0.5 g was extracted with 15 mL 80 % acetone at 4 °C in darkness for 24 h, then suspended by ultrasound for 1 h. After centrifugation at 5000 rpm for 1 min, the supernatant was filtered using a 0.22 µm membrane filter. Chlorophyll level was determined according to the protocol of Arnon. 

Three biological replicates were performed for the above pigments measurement.

### 4.3. RNA Extraction, Library Construction, and Transcriptome Sequencing

Total RNA was extracted using the modified CTAB method. RNase-free DNase I (Tiangen; Beijing, China) was applied to remove contaminating genomic DNA. The RNA purity was determined with a NanoDrop 2000 spectrophotometer (NanoDrop Technologies; Wilmington, DE, USA), and 2% agarose gels were run to verify RNA integrity. The construction of the libraries and the RNA-Seq were performed by the Gene Denovo Corporation (Guangzhou, China). mRNA was enriched with oligo (dT)-rich magnetic beads. Then the enriched mRNA was fragmented into short fragments using fragmentation buffer and reverse transcripted into cDNA with random primers. Second-strand cDNA were synthesized by DNA polymerase I, RNase H, dNTP, and buffer. Then the cDNA fragments were purified with QiaQuick PCR extraction kit, end repaired, poly(A) added, and ligated to Illumina sequencing adapters. The ligation products were size selected by agarose gel electrophoresis, PCR amplified, and sequenced using Illumina HiSeq™ 4000 by Gene Denovo Biotechnology Co. Two biological replicates were used in this section.

### 4.4. De Novo Assembly and Gene Annotation

The raw reads (which has be uploaded in the NCBI’s Sequence Read Archive, SRP095498) were first filtered by removing adaptor sequences, reads containing more than 10% of unknown nucleotides (N) and low quality reads containing more than 50% of low quality (Q-value ≤ 10) bases using Perl script. The remaining high-quality reads were assembled de novo using Trinity 2.1.1. [40]. In assembly, the reads from all three libraries were combined, and then ran Trinity. The longest transcript (from alternative splicing transcripts) was selected as the unigene in this study. 

To annotate the unigenes, we used BLAST + 2.4.0 (http://www.ncbi.nlm.nih.gov/BLAST/) with an E-value threshold of 1 × 10^−5^ to NCBI non-redundant protein (Nr) database released 31 August 2016 (http://www.ncbi.nlm.nih.gov), the Swiss-Prot protein database (http://www.expasy.ch/sprot), the Kyoto Encyclopedia of Genes and Genomes (KEGG) database released 1 July 2016 (http://www.genome.jp/kegg), and the COG/KOG database (http://www.ncbi.nlm.nih.gov/COG). Protein functional annotations could then be obtained according to the best alignment results.

### 4.5. Identification and Analysis of DEGs 

The unigene expression was calculated and normalized to RPKM (Reads Per kb per Million reads) [41]. To identify differentially expressed genes across samples, the edgeR package (http://www.r-project.org/) was used. We identified genes with a fold change ≥ 2 and a false discovery rate (FDR) < 0.05 in a comparison as significant DEGs. The confirmed DEGs were subjected to GO functional enrichment analysis and KEGG pathway analysis. Then, based on KEGG pathway analysis, the DEGs involved in leaf coloration were screened for up/downregulated unigenes, and used for further analysis.

### 4.6. Real-Time Quantitative PCR Analysis

Total RNA was extracted from leaves in S1 and S3 stages, respectively. After treatment with RNase-free DNase I (Tiangen; Beijing, China) according to the user manual, 1 μg of total RNA was reverse-transcribed to first-strand cDNA using the PrimeScript RT reagent kit (Takara; Otsu, Japan). Real-time quantitative PCR (q-PCR) experiments was performed using SYBR Premix Ex Taq (Takara) on ABI 7500 system (Applied Biosystems, Waltham, MA, USA). Primers used are listed in Table S2. Initial denaturation at 95 °C for 10 min, followed by 40 cycles of denaturation for 15 s at 95 °C, annealing for 30 s at 60 °C, and extension for 15 s at 72 °C, followed by a final extension for 15 s at 95 °C. The ubiquitin (Unigene0020903) was used to normalized the q-PCR data [42]. The relative expression levels of genes were calculated using the 2^−ΔΔct^ method. The statistical *p*-value was generated by the paired *t*-test. The statistical significance was defined as *p* < 0.05. Three biological replicates were performed for each gene.

## 5. Conclusions

Our results indicate that *ANS* was the key genes that control the anthocyanin level in tree peony leaf. *POR* was the key gene which regulated the chlorophyll content in tree peony leaf. 

## Figures and Tables

**Table 1:** Putative anthocyanin structural genes identified from differentially expressed genes.

GeneID	log2 Ratio (S3/S1) ^a^	FDR	Annotation
Unigene0004270	6.88	1.17 × 10^−9^	Chalcone isomerase
Unigene0018412	2.90	0.007807	Shikimate hydroxycinnamoyltransferase
Unigene0034354	−1.49	0.000353	Flavanone 3-hydroxylase
Unigene0040930	−1.58	0.000112	Flavonoid 3′-hydroxylase
Unigene0046696	−1.65	0.000548	Dihydroflavonol 4-reductase
Unigene0028008	−3.40	6.79 × 10^−12^	Anthocyanidin synthase
Unigene0036137	−1.06	0.023175	UDP-glycosyltransferase
Unigene0052871	1.90	0.001475	Glucoside glucosyltransferase

^a^ Data were the mean value of two biological replicates.

**Table 2:** Differentially expressed genes in transcription factors of anthocyanin biosynthesis.

GeneID	log2 Ratio (S3/S1) ^a^	FDR	Annotation
Unigene0006988	−10.28	0.002581722	Transcription factor MYB39 [*Aegilops tauschii*]
Unigene0028536	−4.40	3.34 × 10^−12^	MYB-like protein X-like [*Citrus sinensis*]
Unigene0033848	−3.43	3.58 × 10^−6^	MYB domain protein 106 [*Theobroma cacao*]
Unigene0019117	−3.15	5.67 × 10^−5^	MYB domain protein 17 isoform 1 [*Theobroma cacao*]
Unigene0015077	−2.98	1.59 × 10^−8^	MYB-related protein 3R-1-like isoform X1 [*Vitis vinifera*]
Unigene0025015	−2.92	3.02 × 10^−7^	Transcription factor MYB90 [*Vitis vinifera*]
Unigene0015076	−2.77	3.04 × 10^−7^	MYB transcription factor family[*Populus trichocarpa*]
Unigene0017724	−2.73	0.008752272	MYB-related protein 3R-1-like isoform X1 [*Vitis vinifera*]
Unigene0023351	−2.67	0.005438748	MYB-related protein A-like [*Populus euphratica*]
Unigene0003954	−2.57	0.007298376	MYB-related protein 3R-1-like isoform X1 [*Vitis vinifera*]
Unigene0040613	−2.57	0.000168857	MYB family transcription factor APL isoform X2 [*Vitis vinifera*]
Unigene0031865	−2.41	3.11 × 10^−7^	MYB transcription factor family[*Populus trichocarpa*]
Unigene0023350	−2.09	0.006013724	MYB-related protein 3R-1-like [*Prunus mume*]
Unigene0003975	−1.60	0.000354814	Transcription factor MYB1R1-like [*Citrus sinensis*]
Unigene0031901	−1.19	0.032182662	MYB DNA-binding domain protein [*Theobroma cacao*]
Unigene0017529	1.10	0.010140177	MYB transcription factor family [*Populus trichocarpa*]
Unigene0022785	1.32	0.041043118	Transcription factor MYB44-like [*Vitis vinifera*]
Unigene0030933	1.48	0.01321785	MYB-related protein 308-like [*Populus euphratica*]
Unigene0003846	1.57	0.02700583	MYB transcription factor family [*Populus trichocarpa*]
Unigene0003228	2.00	0.009708022	MYB-like protein J [*Malus domestica*]
Unigene0021304	2.21	0.028027338	Transcription factor MYB59 [*Vitis vinifera*]
Unigene0009655	2.25	0.000336502	MYB transcription factor family [*Populus trichocarpa*]
Unigene0018495	2.27	0.000636996	Transcription factor MYB82 [*Jatropha curcas*]
Unigene0016379	2.49	0.000248643	MYB family transcription factor APL [*Prunus mume*]
Unigene0013255	2.87	0.002952733	R2R3 MYBA6 transcription factor splice variant 1 [*Vitis vinifera*]
Unigene0018284	3.07	0.002052337	MYB family transcription factor At1g14600 [*Vitis vinifera*]
Unigene0029454	3.13	6.71 × 10^−5^	MYB-like transcription factor [*Betula luminifera*]
Unigene0017856	−6.92	2.88 × 10^−20^	Transcription factor bHLH94-like [*Nelumbo nucifera*]
Unigene0011325	−3.98	0.001209742	bHLH family protein [*Populus trichocarpa*]
Unigene0011327	−3.79	0.000339255	bHLH family protein [*Populus trichocarpa*]
Unigene0001264	−3.48	0.000243391	bHLH family protein [*Populus trichocarpa*]
Unigene0023425	−3.26	6.47 × 10^−5^	Transcription factor bHLH118-like [*Jatropha curcas*]
Unigene0013501	−2.60	0.033069525	Transcription factor bHLH93 [*Vitis vinifera*]
Unigene0026438	−2.44	0.000225777	Transcription factor bHLH70 isoform X2 [*Vitis vinifera*]
Unigene0030075	−2.32	0.000257132	Transcription factor bHLH30-like [*Jatropha curcas*]
Unigene0031959	−2.14	0.000135867	Transcription factor bHLH66-like isoform X4 [*Vitis vinifera*]
Unigene0041894	−1.94	5.05 × 10^−5^	Transcription factor bHLH35-like [*Pyrus x bretschneideri*]
Unigene0041893	−1.90	0.00691905	Transcription factor bHLH35-like [*Pyrus x bretschneideri*]
Unigene0033453	−1.66	0.000163627	Myc anthocyanin regulatory protein isoform X1 [*Vitis vinifera*]
Unigene0009061	−1.60	0.011318569	Transcription factor bHLH104-like isoform X1 [*Citrus sinensis*]
Unigene0000218	−1.28	0.026379909	Transcription factor bHLH49 isoform X1 [*Vitis vinifera*]
Unigene0037994	−1.22	0.03284377	Transcription factor bHLH145-like [*Prunus mume*]
Unigene0033727	1.06	0.015085073	Transcription factor bHLH63 [*Vitis vinifera*]
Unigene0014388	1.18	0.03179475	Transcription factor bHLH51 [*Vitis vinifera*]
Unigene0020369	1.79	0.00037921	Transcription factor bHLH149 [*Solanum lycopersicum*]
Unigene0023437	1.81	0.00014609	Transcription factor bHLH63 [*Prunus mume*]
Unigene0027343	1.88	0.000282396	Transcription factor bHLH61 isoform 2 [*Theobroma cacao*]
Unigene0032390	−5.15	6.23 × 10^−17^	F-box/WD repeat-containing 10 [*Gossypium arboreum*]
Unigene0019877	−4.98	1.75 × 10^−6^	WD repeat-containing 62 [*Gossypium arboreum*]
Unigene0052972	−3.88	8.77 × 10^−8^	WD repeat-containing protein 76 isoform X2 [*Jatropha curcas*]
Unigene0017625	−3.76	0.000361154	F-box/WD repeat-containing protein sel-10 [*Jatropha curcas*]
Unigene0038354	−3.59	1.16 × 10^−14^	WD repeat-containing protein 76 isoform X1 [*Jatropha curcas*]
Unigene0038352	−3.35	6.74 × 10^−8^	WD repeat-containing protein 76 isoform X2 [*Jatropha curcas*]
Unigene0038982	−3.33	5.71 × 10^−10^	WD repeat and HMG-box DNA-binding protein 1 [*Vitis vinifera*]
Unigene0004606	−3.25	1.42 × 10^−6^	WD repeat-containing protein 76 isoform X1 [*Jatropha curcas*]
Unigene0016452	−2.38	2.55 × 10^−6^	WD-40 repeat-containing protein MSI3 [*Vitis vinifera*]
Unigene0038353	−2.35	0.000579262	WD repeat-containing protein 76 [*Gossypium raimondii*]
Unigene0038355	−2.28	0.004815745	WD repeat-containing protein 76 isoform X2 [*Jatropha curcas*]
Unigene0033696	−2.24	6.56 × 10^−7^	Transducin/WD40 repeat-like superfamily protein [*Theobroma cacao*]
Unigene0033696	−2.24	6.56 × 10^−7^	Transducin/WD40 repeat-like superfamily protein [*Theobroma cacao*]
Unigene0031235	−2.22	1.05 × 10^−8^	WD-40 repeat-containing protein MSI4-like [*Eucalyptus grandis]*
Unigene0017354	−2.05	0.000604273	WD repeat-containing protein 5-like [*Nelumbo nucifera*]
Unigene0041973	−1.94	4.57 × 10^−5^	WD repeat-containing protein 43 isoform X1 [*Vitis vinifera*]
Unigene0011781	−1.86	0.003509861	Glutamate-rich WD repeat-containing protein 1 [*Nelumbo nucifera*]
Unigene0046703	−1.71	0.023069191	WD repeat-containing 6 [*Gossypium arboreum*]
Unigene0041702	−1.68	0.002347404	WD repeat-containing protein 3 [*Vitis vinifera*]
Unigene0018836	−1.63	0.007290383	WD repeat-containing protein 82 [*Jatropha curcas*]
Unigene0024647	−1.61	2.61 × 10^−5^	Transducin/WD40 repeat-like superfamily protein [*Theobroma cacao*]
Unigene0028464	−1.47	0.007517559	WD repeat-containing protein [*Gossypium arboreum*]
Unigene0041538	−1.39	0.0043636	WD repeat-containing protein 87-like [*Cicer arietinum*]
Unigene0030123	−1.34	0.001589388	WD-40 repeat-containing protein MSI1 [*Vitis vinifera*]
Unigene0051975	−1.32	0.002923971	WD repeat-containing protein 61 [*Nelumbo nucifera*]
Unigene0031526	−1.18	0.018270378	Katanin p80 WD40 repeat-containing subunit B1 homolog isoform X2 [*Vitis vinifera*]
Unigene0039012	−1.13	0.010137359	WD repeat-containing protein 70 [*Vitis vinifera*]
Unigene0032745	−1.12	0.014302649	Transducin/WD40 repeat-like superfamily protein [*Theobroma cacao*]
Unigene0045126	−1.08	0.031686906	WD40 protein [*Paeonia suffruticosa*]
Unigene0049600	1.24	0.010794516	Transducin/WD40 repeat-like superfamily protein [*Theobroma cacao*]
Unigene0019158	1.62	0.014228592	WD repeat-containing protein LWD1-like [*Gossypium raimondii*]
Unigene0029541	1.84	0.000911988	WD repeat-containing 70 [*Gossypium arboreum*]
Unigene0015065	2.21	3.62 × 10^−6^	F-box/WD repeat-containing protein [*Medicago truncatula*]
Unigene0051483	2.58	0.008905399	Transducin/WD40 repeat-like superfamily protein [*Theobroma cacao*]
Unigene0033466	3.91	5.28 × 10^−6^	Transducin/WD40 repeat-like superfamily protein [*Theobroma cacao*]
Unigene0033465	5.08	1.24 × 10^−5^	Transducin/WD40 repeat-like superfamily protein [*Theobroma cacao*]

^a^ Data were the mean value of two biological replicates.

**Table 3:** Putative chlorophyll genes identified from differentially expressed genes.

GeneID	log2 Ratio (S3/S2) ^a^	FDR	Annotation
Unigene0031269	1.94	0.03382	Mg-protoporphyrinogen IX monomethylester
Unigene0022913	1.02	0.047351	Divinyl reductase
Unigene0036679	1.03	0.033455	Chlorophyllide a oxygenase
Unigene0045551	−1.42	0.009836	Glutamyl-tRNA synthetase
Unigene0032398	1.09	0.02502	Protochlorophyllide oxidoreductase
Unigene0039869	−5.47	6.00 × 10^−16^	Geranylgeranyl reductase
Unigene0047139	1.40	0.003807	Pheophorbide a oxygenase
Unigene0026448	1.32	0.019098	Protoheme ferro-lyase

^a^ Data were the mean value of two biological replicates.

**Table 4:** Putative carotenoids genes identified from differentially expressed genes.

GeneID	log2 Ratio (S3/S1) ^a^	FDR	Annotation
Unigene0029992	2.07	2.10 × 10^−7^	Phytoene synthase 2, chloroplastic-like
Unigene0030040	1.77	3.96 × 10^−5^	Phytoene desaturase, partial
Unigene0025170	2.28	4.78 × 10^−5^	Beta-carotene isomerase D27
Unigene0039699	1.71	3.42 × 10^−5^	Zeta-carotene desaturase
Unigene0013558	1.99	3.48 × 10^−7^	Violaxanthin de-epoxidase, chloroplastic
Unigene0023761	1.08	0.014272	Lycopene beta cyclase, chloroplastic
Unigene0016440	1.38	0.000987	Lycopene epsilon cyclase
Unigene0032549	2.55	0.013492	Beta-carotene hydroxylase, partial
Unigene0027491	1.60	5.36 × 10^−5^	Zeaxanthin epoxidase, partial
Unigene0051809	2.88	2.71 × 10^−13^	Carotenoid cleavage dioxygenase

^a^ Data were the mean value of two biological replicates.

## Supplementary Materials

Supplementary materials are available online.
