# Supplementary material for: Transcriptomic Analysis of Leaf in Tree Peony Reveals Differentially Expressed Pigments Genes
Source: Molecules. 2017 Feb 20;22(2):324. doi: 10.3390/molecules22020324 (PMC6155769; doi:10.3390/molecules22020324)
Supplement: Supplementary file 1 [file molecules-22-00324-s001.pdf]

Supplementary Materials: Transcriptomic Analysis of Leaf in Tree Peony Reveals Differentially Expressed Pigments Genes

Jianrang Luo, Lixin Niu, Qianqian Shi and Yanlong Zhang

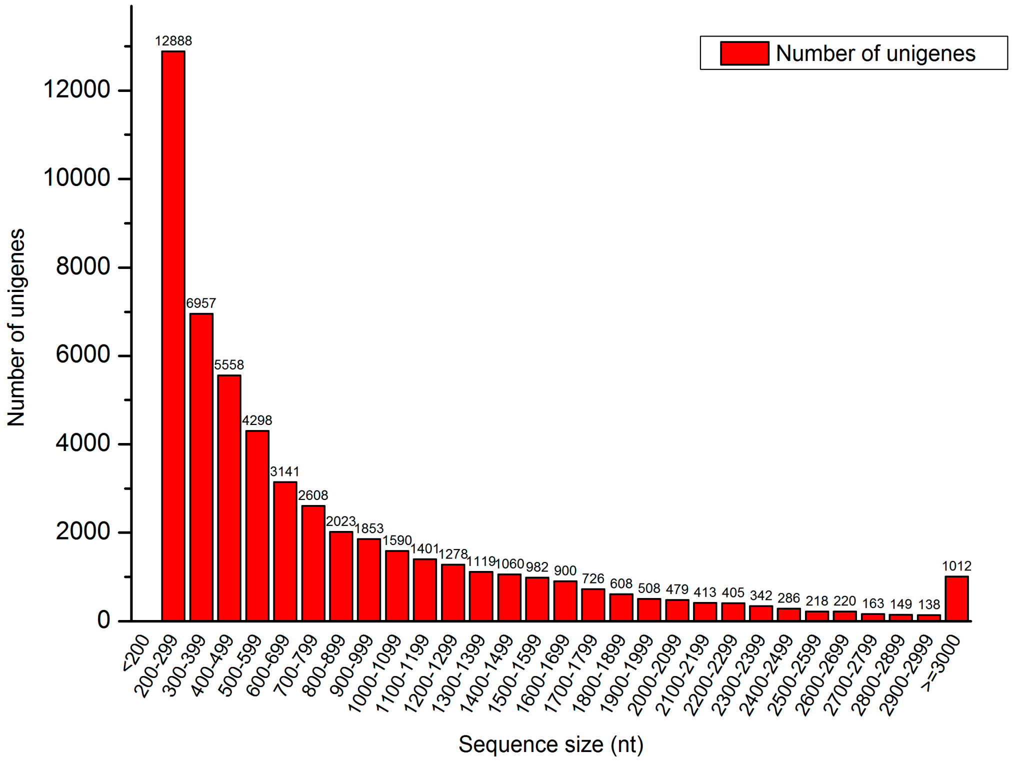

Figure S1. The size distribution of unigenes.

Table S1. KEGG pathway annotation of differentially expressed genes in S1 vs S3.

No.	Pathway	DEGs genes with pathway annotation (1436)	All genes with pathway annotation (5898)	Pvalue	Qvalue	Pathway ID
1	Ribosome	195 (13.58%)	510 (8.65%)	0	0	ko03010
2	DNA replication	43 (2.99%)	80 (1.36%)	0	0.000001	ko03030
3	Photosynthesis	33 (2.3%)	74 (1.25%)	0.0001	0.004047	ko00195
4	Mismatch repair	28 (1.95%)	62 (1.05%)	0.000256	0.007746	ko03430
5	Glycine, serine and threonine metabolism	27 (1.88%)	66 (1.12%)	0.002079	0.046259	ko00260
6	Monoterpenoid biosynthesis	8 (0.56%)	12 (0.2%)	0.002294	0.046259	ko00902
7	Proteasome	26 (1.81%)	64 (1.09%)	0.002803	0.048456	ko03050
8	Ribosome biogenesis in eukaryotes	53 (3.69%)	157 (2.66%)	0.004505	0.068133	ko03008
9	Base excision repair	24 (1.67%)	62 (1.05%)	0.008179	0.105087	ko03410
10	Phenylpropanoid biosynthesis	44 (3.06%)	130 (2.2%)	0.008685	0.105087	ko00940
11	Spliceosome	91 (6.34%)	302 (5.12%)	0.010883	0.119711	ko03040

12	Homologous recombination	27 (1.88%)	75 (1.27%)	0.015394	0.155219	ko03440
13	Limonene and pinene degradation	6 (0.42%)	10 (0.17%)	0.017222	0.160293	ko00903
14	Alanine, aspartate and glutamate metabolism	18 (1.25%)	47 (0.8%)	0.023012	0.182775	ko00250
15	Brassinosteroid biosynthesis	8 (0.56%)	16 (0.27%)	0.023092	0.182775	ko00905
16	Photosynthesis - antenna proteins	9 (0.63%)	19 (0.32%)	0.024169	0.182775	ko00196
17	Nucleotide excision repair	31 (2.16%)	92 (1.56%)	0.026538	0.188889	ko03420
18	Glyoxylate and dicarboxylate metabolism	29 (2.02%)	86 (1.46%)	0.030894	0.207678	ko00630
19	Cutin, suberine and wax biosynthesis	15 (1.04%)	39 (0.66%)	0.034881	0.22214	ko00073
20	Nitrogen metabolism	10 (0.7%)	24 (0.41%)	0.046105	0.269627	ko00910
21	Carbon fixation in photosynthetic organisms	31 (2.16%)	96 (1.63%)	0.046795	0.269627	ko00710
22	Purine metabolism	62 (4.32%)	212 (3.59%)	0.055633	0.305983	ko00230
23	Biotin metabolism	10 (0.7%)	25 (0.42%)	0.060611	0.306626	ko00780
24	Cysteine and methionine metabolism	37 (2.58%)	120 (2.03%)	0.061388	0.306626	ko00270
25	Pyrimidine metabolism	48 (3.34%)	161 (2.73%)	0.063352	0.306626	ko00240
26	Pentose and glucuronate interconversions	21 (1.46%)	63 (1.07%)	0.06723	0.31288	ko00040
27	Isoflavonoid biosynthesis	3 (0.21%)	5 (0.08%)	0.096663	0.433192	ko00943
28	Cyanoamino acid metabolism	21 (1.46%)	66 (1.12%)	0.10282	0.441518	ko00460
29	Stilbenoid, diarylheptanoid and gingerol biosynthesis	9 (0.63%)	24 (0.41%)	0.105818	0.441518	ko00945
30	Carotenoid biosynthesis	13 (0.91%)	38 (0.64%)	0.111346	0.449096	ko00906
31	Glycolysis / Gluconeogenesis	42 (2.92%)	147 (2.49%)	0.133893	0.522614	ko00010
32	Starch and sucrose metabolism	63 (4.39%)	229 (3.88%)	0.145001	0.548287	ko00500
33	Monobactam biosynthesis	4 (0.28%)	9 (0.15%)	0.153581	0.5594	ko00261
34	Histidine metabolism	7 (0.49%)	19 (0.32%)	0.157187	0.5594	ko00340
35	Circadian rhythm - plant	14 (0.97%)	44 (0.75%)	0.162328	0.56119	ko04712
36	Vitamin B6 metabolism	6 (0.42%)	16 (0.27%)	0.172499	0.579788	ko00750
37	Fatty acid elongation	11 (0.77%)	34 (0.58%)	0.184534	0.603477	ko00062
38	Degradation of aromatic compounds	5 (0.35%)	13 (0.22%)	0.189681	0.603983	ko01220
39	One carbon pool by folate	8 (0.56%)	24 (0.41%)	0.210419	0.652838	ko00670
40	Valine, leucine and isoleucine degradation	17 (1.18%)	58 (0.98%)	0.228682	0.691763	ko00280
41	Ascorbate and aldarate metabolism	13 (0.91%)	44 (0.75%)	0.258604	0.74642	ko00053
42	Flavonoid biosynthesis	9 (0.63%)	29 (0.49%)	0.259088	0.74642	ko00941
43	Plant hormone signal transduction	59 (4.11%)	226 (3.83%)	0.288474	0.811752	ko04075
44	Fructose and mannose metabolism	23 (1.6%)	85 (1.44%)	0.317098	0.872018	ko00051
45	Biosynthesis of amino acids	73 (5.08%)	286 (4.85%)	0.339454	0.896371	ko01230
46	RNA transport	69 (4.81%)	270 (4.58%)	0.340769	0.896371	ko03013
47	Anthocyanin biosynthesis	2 (0.14%)	5 (0.08%)	0.353416	0.909857	ko00942
48	Tryptophan metabolism	8 (0.56%)	28 (0.47%)	0.368838	0.929779	ko00380
49	Lysine biosynthesis	5 (0.35%)	17 (0.29%)	0.401105	0.952562	ko00300
50	Zeatin biosynthesis	6 (0.42%)	21 (0.36%)	0.405649	0.952562	ko00908
51	Arginine biosynthesis	10 (0.7%)	37 (0.63%)	0.412908	0.952562	ko00220

52	Phenylalanine, tyrosine and tryptophan biosynthesis	14 (0.97%)	53 (0.9%)	0.413913	0.952562	ko00400
53	Flavone and flavonol biosynthesis	1 (0.07%)	2 (0.03%)	0.427697	0.952562	ko00944
54	Terpenoid backbone biosynthesis	16 (1.11%)	62 (1.05%)	0.442377	0.952562	ko00900
55	Pentose phosphate pathway	16 (1.11%)	62 (1.05%)	0.442377	0.952562	ko00030
56	Synthesis and degradation of ketone bodies	2 (0.14%)	6 (0.1%)	0.450553	0.952562	ko00072
57	Sesquiterpenoid and triterpenoid biosynthesis	5 (0.35%)	18 (0.31%)	0.455329	0.952562	ko00909
58	Phagosome	24 (1.67%)	95 (1.61%)	0.4566	0.952562	ko04145
59	Biosynthesis of unsaturated fatty acids	13 (0.91%)	51 (0.86%)	0.47809	0.980491	ko01040
60	Protein processing in endoplasmic reticulum	79 (5.5%)	322 (5.46%)	0.490509	0.989192	ko04141
61	Galactose metabolism	18 (1.25%)	74 (1.25%)	0.547051	1	ko00052
62	Lysine degradation	10 (0.7%)	41 (0.7%)	0.557764	1	ko00310
63	N-Glycan biosynthesis	12 (0.84%)	50 (0.85%)	0.577537	1	ko00510
64	Tyrosine metabolism	14 (0.97%)	59 (1%)	0.594491	1	ko00350
65	Carbon metabolism	78 (5.43%)	327 (5.54%)	0.606832	1	ko01200
66	Arginine and proline metabolism	12 (0.84%)	51 (0.86%)	0.608136	1	ko00330
67	Linoleic acid metabolism	4 (0.28%)	17 (0.29%)	0.623798	1	ko00591
68	Nicotinate and nicotinamide metabolism	5 (0.35%)	22 (0.37%)	0.651118	1	ko00760
69	Caffeine metabolism	1 (0.07%)	4 (0.07%)	0.672541	1	ko00232
70	Fatty acid biosynthesis	11 (0.77%)	49 (0.83%)	0.675484	1	ko00061
71	Fatty acid metabolism	22 (1.53%)	98 (1.66%)	0.707545	1	ko01212
72	Diterpenoid biosynthesis	4 (0.28%)	20 (0.34%)	0.754873	1	ko00904
73	Glutathione metabolism	20 (1.39%)	92 (1.56%)	0.757861	1	ko00480
74	Fatty acid degradation	12 (0.84%)	57 (0.97%)	0.765751	1	ko00071
75	Phenylalanine metabolism	9 (0.63%)	45 (0.76%)	0.80188	1	ko00360
76	Tropane, piperidine and pyridine alkaloid biosynthesis	5 (0.35%)	28 (0.47%)	0.84765	1	ko00960
77	Isoquinoline alkaloid biosynthesis	5 (0.35%)	28 (0.47%)	0.84765	1	ko00950
78	Other glycan degradation	6 (0.42%)	33 (0.56%)	0.849645	1	ko00511
79	Non-homologous end-joining	3 (0.21%)	18 (0.31%)	0.851219	1	ko03450
80	Steroid biosynthesis	7 (0.49%)	38 (0.64%)	0.852729	1	ko00100
81	RNA polymerase	12 (0.84%)	63 (1.07%)	0.873266	1	ko03020
82	Pyruvate metabolism	19 (1.32%)	96 (1.63%)	0.880455	1	ko00620
83	Arachidonic acid metabolism	3 (0.21%)	20 (0.34%)	0.898131	1	ko00590
84	Protein export	12 (0.84%)	65 (1.1%)	0.898781	1	ko03060
85	Taurine and hypotaurine metabolism	2 (0.14%)	15 (0.25%)	0.911593	1	ko00430
86	Plant-pathogen interaction	49 (3.41%)	235 (3.98%)	0.913604	1	ko04626
87	Butanoate metabolism	3 (0.21%)	21 (0.36%)	0.916187	1	ko00650
88	Inositol phosphate metabolism	13 (0.91%)	72 (1.22%)	0.921633	1	ko00562
89	Phosphatidylinositol signaling system	13 (0.91%)	72 (1.22%)	0.921633	1	ko04070
90	Glycerolipid metabolism	10 (0.7%)	58 (0.98%)	0.92704	1	ko00561
91	Sulfur metabolism	5 (0.35%)	33 (0.56%)	0.931635	1	ko00920

92	Regulation of autophagy	8 (0.56%)	49 (0.83%)	0.936286	1	ko04140
93	Glycosphingolipid biosynthesis - globo series	1 (0.07%)	10 (0.17%)	0.938739	1	ko00603
94	2-Oxocarboxylic acid metabolism	11 (0.77%)	66 (1.12%)	0.950982	1	ko01210
95	alpha-Linolenic acid metabolism	9 (0.63%)	56 (0.95%)	0.951535	1	ko00592
96	Thiamine metabolism	2 (0.14%)	18 (0.31%)	0.955467	1	ko00730
97	beta-Alanine metabolism	7 (0.49%)	47 (0.8%)	0.960616	1	ko00410
98	Amino sugar and nucleotide sugar metabolism	28 (1.95%)	150 (2.54%)	0.962091	1	ko00520
99	RNA degradation	34 (2.37%)	179 (3.03%)	0.965533	1	ko03018
100	Citrate cycle (TCA cycle)	12 (0.84%)	75 (1.27%)	0.971161	1	ko00020
101	Peroxisome	21 (1.46%)	121 (2.05%)	0.975849	1	ko04146
102	Valine, leucine and isoleucine biosynthesis	2 (0.14%)	21 (0.36%)	0.978031	1	ko00290
103	mRNA surveillance pathway	31 (2.16%)	170 (2.88%)	0.978552	1	ko03015
104	Sphingolipid metabolism	4 (0.28%)	34 (0.58%)	0.979818	1	ko00600
105	Selenocompound metabolism	2 (0.14%)	23 (0.39%)	0.98641	1	ko00450
106	Aminoacyl-tRNA biosynthesis	11 (0.77%)	76 (1.29%)	0.988119	1	ko00970
107	Glycosaminoglycan degradation	1 (0.07%)	16 (0.27%)	0.988562	1	ko00531
108	Propanoate metabolism	5 (0.35%)	44 (0.75%)	0.990827	1	ko00640
109	Folate biosynthesis	2 (0.14%)	25 (0.42%)	0.991644	1	ko00790
110	Ubiquinone and other terpenoid-quinone biosynthesis	6 (0.42%)	58 (0.98%)	0.998054	1	ko00130
111	Ether lipid metabolism	4 (0.28%)	46 (0.78%)	0.998374	1	ko00565
112	Glycosylphosphatidylinositol(GPI)-anchor biosynthesis	2 (0.14%)	32 (0.54%)	0.998533	1	ko00563
113	Pantothenate and CoA biosynthesis	3 (0.21%)	41 (0.7%)	0.998964	1	ko00770
114	SNARE interactions in vesicular transport	4 (0.28%)	52 (0.88%)	0.999573	1	ko04130
115	Endocytosis	34 (2.37%)	229 (3.88%)	0.99988	1	ko04144
116	ABC transporters	8 (0.56%)	86 (1.46%)	0.99991	1	ko02010
117	Oxidative phosphorylation	23 (1.6%)	173 (2.93%)	0.999911	1	ko00190
118	Porphyrin and chlorophyll metabolism	4 (0.28%)	63 (1.07%)	0.999966	1	ko00860
119	Ubiquitin mediated proteolysis	31 (2.16%)	224 (3.8%)	0.999973	1	ko04120
120	Glycerophospholipid metabolism	11 (0.77%)	116 (1.97%)	0.99999	1	ko00564
121	Basal transcription factors	3 (0.21%)	77 (1.31%)	1	1	ko03022

Table S2. Primers used for qRT-PCR analysis.

Genes	Primer
Unigene0004270-F	CACTTAGGAGCGAGCGACTT
Unigene0004270-R	CGCAGGAGTGAGAGGATTGG
Unigene0018412-F	GGAGTCCCGAAATAGGTTGC
Unigene0018412-R	AACGGCTTTGACACAAGGTC
Unigene0013558-F	TGCGCACTCGTTGAACTCAT
Unigene0013558-R	TGCTGCAAATGTTGCCTGTC
Unigene0016440-F	TGCCTTACTTGGTCCTCACG
Unigene0016440-R	TTCACTTCCTGCAGTAGCCC
Unigene0022913-F	AGCCAAATCCTGCTCGCTAA

Unigene0022913-R	TAAGAGCTTGGGTGGGCAAG
Unigene0023761-F	AGTCCAAGCTGCCGTAGTTC
Unigene0023761-R	AAGCAACTTGGTAACCCGGA
Unigene0025170-F	ATCGATGGTGTCACGCAACT
Unigene0025170-R	CATGATGGGCGCTGGAAATG
Unigene0027491-F	TTATCGCACCAACCTCCGAA
Unigene0027491-R	ACGCCTTCCACAATGAACCA
Unigene0028008-F	CAACCAGCGAATACGCCAAG
Unigene0028008-R	CTTCCATGCCACCGACTTCT
Unigene0029992-F	TGCACTGGGCTAACCTTTCA
Unigene0029992-R	TGGGGGAGGCTTTTCAATGT
Unigene0030040-F	GCGGATCAGAGCAAGGCTAA
Unigene0030040-R	AAGGGACGACATGGTTCACA
Unigene0031269-F	TTTTGTGTTGAGCCCAATGCC
Unigene0031269-R	TCCTGCAGGTTTACGTGACAA
Unigene0032398-F	ACACTTTACGCGCCTTCTCT
Unigene0032398-R	ACTACCCGTCCAAGCGTATG
Unigene0032549-F	TCTGTTTTGGCGCTGGTTTG
Unigene0032549-R	GGACATTAGCAACGGGTCCT
Unigene0034354-F	CCGAAATGTCCTCAACCGGA
Unigene0034354-R	GCCACCCACCTGATCTTGAA
Unigene0036679-F	TGGAAAGCCTGGATGTGTCC
Unigene0036679-R	ATGCGCCCTTCATTCACAGA
Unigene0039699-F	GATTTCCAGTCGGAGCACCA
Unigene0039699-R	TGCAAGTGCCACAGCATTTC
Unigene0040930-F	CAAATGCACTGGCAAGGGTC
Unigene0040930-R	TGAATTTGTCGGCCTTGGGA
Unigene0046696-F	CCATGTCACTCCAGCAGGTT
Unigene0046696-R	TGTGCGGAGAGTCGTTTTCA
Unigene0051809-F	GACGAGCTTCCACCAACAGA
Unigene0051809-R	ACTGTGGGTTAGGGCCATTG
Unigene0020903-F	GAAAAGGCCAGTTGCTTCGG
Unigene0020903-R	AGAAATGGAGTGGGCACCTG
